# Efficacy of COVID-19 Convalescent Plasma Based on Antibody Concentration

**DOI:** 10.1155/2022/7992927

**Published:** 2022-09-17

**Authors:** Wesley V Cain, Anne M Sill, Vinod Solipuram, John J Weiss, Carole B Miller, Peter F Jelsma

**Affiliations:** ^1^The Department of Medicine, University of Tennessee Health Science Center, Ascension Saint Thomas Hospital Midtown, Nashville, TN, USA; ^2^Ascension Saint Agnes, Baltimore, MD, USA; ^3^Allermetrix Inc, Franklin, TN, USA; ^4^Department of Medicine, Ascension Saint Agnes Hospital, Baltimore, MD, USA; ^5^Ascension Saint Agnes Hospital, Baltimore, MD, USA; ^6^Ascension Saint Thomas Hospital, Nashville, TN, USA

## Abstract

**Background:**

Convalescent plasma obtained from individuals who have recovered from severe acute respiratory syndrome coronavirus 2 (SARS-CoV-2) contains neutralizing antibodies to the virus and has been frequently used as a treatment in hospitalized patients with severe COVID-19.

**Methods:**

We conducted a retrospective, observational cohort study involving 96 hospitalized patients with severe COVID-19 who were allocated in a 1 : 1 ratio to having received either high antibody concentration convalescent plasma or low antibody concentration convalescent plasma. Quantitative measurements of IgG to the receptor-binding domain (RBD), the S1 subunit of the spike protein, and the SARS-CoV-2 nucleocapsid (N) protein were determined from donor plasma samples. The primary outcome was all-cause mortality within 30 days following convalescent plasma administration in regard to each of the three antibody domains.

**Results:**

Within the nucleocapsid antibody domain, death occurred in 22.2% of patients in the low antibody concentration group versus 23.5% in the high antibody concentration group (*p*=0.88). Within the RBD antibody domain, death occurred in 22.9% of patients in both the low and the high antibody concentration groups (*p*=1.0). Within the S1 subunit antibody domain, death occurred in 27.1% of patients in the low antibody concentration group versus 18.8% in the high antibody concentration group (*p*=0.33).

**Conclusions:**

No significant differences were observed between low and high concentration convalescent plasma in regard to overall mortality at 30 days, hospital length of stay, number of ventilator days, and subsequent receipt of invasive mechanical ventilation in patients who were previously not receiving mechanical ventilation. *Trial Registration*. This study was not associated with a clinical trial due to the retrospective nature of study design.

## 1. Introduction

Outbreak of novel coronavirus COVID-19 was first reported at the end of 2019 in Wuhan, China, which has since emerged as a pandemic affecting millions of people globally. COVID-19, which is caused by severe acute respiratory syndrome coronavirus 2 (SARS-CoV-2), has affected approximately 85.8 million people in the United States as of 6/14/2022 with an observed case fatality rate of 1.2% [1]. SARS-CoV-2 infection in humans can present variably ranging from asymptomatic carriers to acute respiratory distress syndrome (ARDS). It mainly affects the respiratory system in humans causing pneumonia, but detrimental effects on several other organ systems have also been reported and the transmission is predominantly by respiratory droplets [2].

Since the onset of the pandemic, many clinical trials have been underway to evaluate therapies for treatment of COVID-19. One therapy that has been given to hospitalized patients with severe COVID-19 infection is convalescent plasma. This refers to plasma obtained from donors who have recovered from COVID-19 which contains antibodies to SARS-CoV-2 that may help suppress the virus and modify the inflammatory response [3]. Humoral immunity is a crucial component of the immune response to COVID-19. Antibodies to SARS-CoV-2 are detectable at a mean of 13 days after symptom onset, but neutralizing titers do not peak until day 23 with a wide variation in both the timing of seroconversion and the peak antibody concentrations between individuals infected with the virus [4]. COVID-19 convalescent plasma contains neutralizing, polyclonal antibodies to several viral proteins of SARS-CoV-2, including antibodies directed against the receptor-binding domain (RBD) of the spike protein of SARS-CoV-2, the S1 subunit of the spike protein, and the SARS-CoV-2 nucleocapsid (N) protein. These neutralizing antibodies from convalescent plasma serve as passive immunity until a disease-specific immune response can be elicited from the host. Several nonrandomized trials done in the past for SARS (severe acute respiratory syndrome), MERS (Middle East respiratory syndrome), influenza A (H1N1), avian influenza (H5N1), and Ebola have claimed efficacy of convalescent plasma, though data from randomized, controlled trials are lacking [5–8].

Convalescent plasma was initially provided to more than 100,000 patients in the United States diagnosed with COVID-19 through Mayo Clinic's Expanded Access Program (EAP). Both the Food and Drug Administration (FDA) and the Mayo Clinic performed retrospective, indirect evaluations of efficacy by using the Mayo Clinic EAP data, hypothesizing that patients who received plasma units with higher titers of SARS-CoV-2 neutralizing antibodies would have better clinical outcomes than those who received plasma units with lower antibody titers [9]. The results of their analyses suggested that convalescent plasma with high antibody titers was associated with a lower risk of death compared to transfusion of plasma with lower antibody levels in nonintubated patients, particularly when administered within 72 hours of COVID-19 diagnosis. Based on these findings, convalescent plasma was given emergency use authorization (EUA) by the FDA for treatment of hospitalized patients with COVID-19 on August 23, 2020. However, on February 4, 2021, the FDA revised the convalescent plasma EUA to limit the authorization to high-titer COVID-19 convalescent plasma and only for the treatment of hospitalized patients with COVID-19 early in the disease course or in hospitalized patients who have impaired humoral immunity [10].

The efficacy of convalescent plasma is unclear, given mixed results among the studies. The Randomised Evaluation of COVID-19 Therapy (RECOVERY) trial is an open-label, randomized controlled platform trial evaluating potential treatments for COVID-19, including convalescent plasma. In the convalescent plasma portion of the trial, 11,558 patients were randomized to receive either high-titer convalescent plasma or usual care. Their results showed no significant difference in 28-day mortality between the two arms of the study (24% vs. 24%; rate ratio 1.00; 95% CI, 0.93–1.07) [11]. In addition, there was no difference in the proportion of patients discharged within 28 days and no difference between the percentage of patients who progressed to invasive mechanical ventilation in previously nonventilated patients. Several other randomized clinical trials have also evaluated the efficacy of convalescent plasma for the treatment of hospitalized patients with COVID-19, all of which were unable to demonstrate efficacy [12–15].

Further research on COVID-19 convalescent plasma would be extremely useful to understand the efficacy of convalescent plasma based on antibody levels. In this retrospective cohort study, we are testing the hypothesis that administration of convalescent plasma with high antibody concentration is associated with a lower mortality rate at 30 days compared to administration with low antibody concentration in patients hospitalized with severe COVID-19. We also aim to address other outcomes such as hospital length of stay, subsequent receipt of mechanical ventilation after transfusion, and number of ventilator days in patients receiving convalescent plasma.

## 2. Methods

### 2.1. Study Design and Participants

This study is a retrospective, observational, nonrandomized cohort study involving 3 medical centers in the United States. Recipients of donor plasma were admitted between April 1, 2020, and July 31, 2020. Participants in the study were allocated in a 1 : 1 ratio to having received either high antibody concentration convalescent plasma or low antibody concentration convalescent plasma. The trial protocol was approved by the institutional review board. Patients or their representatives provided written consent for the treatment.

### 2.2. Donor Selection and Laboratory Analysis

Donor plasma from individuals who recovered from COVID-19 was collected by Blood Assurance, a full-service regional blood center based in Chattanooga, TN. Recovered patients were able to donate plasma through Blood Assurance if they qualified for one of two categories: category (1) patient qualified had a documented positive nasopharyngeal COVID-19 test and had been symptom-free for greater than or equal to 14 days; category (2) patient never tested for COVID-19 and had been symptom-free for greater than 14 days. Those individuals in category 2 then had to test positive for COVID-19 antibodies using the Abbott ARCHITECT SARS-CoV-2 IgG immunoassay. Samples testing positive then had to test positive using the Diazyme DZ-Lite SARS-CoV-2 IgG assay. This ensured that antibodies against both the nucleocapsid protein and the S1 subunit of the spike protein were being accounted for in the testing.

Samples of this donor plasma were sent to Allermetrix Inc. (Franklin, TN), a College of American Pathology- (CAP-) certified allergy specialty laboratory. Allermetrix Inc. has developed a quantitative method for measuring IgG and IgM to the receptor-binding domain (RBD) of the spike protein of SARS-CoV-2, the S1 subunit of the spike protein, and the SARS-CoV-2 nucleocapsid (N) protein that has been validated for use in plasma and serum. Quantitation offers better accuracy and precision than titering. Antibody responses are polyclonal and there may be several antibodies that will react with each of the target antigens. Quantitation uses a relatively low dilution of serum compared to titers and measures all the antibody specificities present while titers only measure the most abundant antibodies. Titering precision is generally ±one dilution which in precision terms is about a 100% coefficient of variation. The quantitative assay coefficient of variation is about 15%, which is nearly an order of magnitude more precise.

### 2.3. Inclusion and Exclusion Criteria

Hospitalized patients were eligible for the study if they were greater than 18 years of age and diagnosed with COVID-19 by a reverse-transcriptase–polymerase chain reaction (RT-PCR) assay that was positive for SARS-CoV-2 and received convalescent plasma as part of their treatment. Additionally, meeting one of the following severity criteria was required: (1) less than 93% oxygen saturation on ambient air at rest, (2) partial pressure of arterial oxygen (PaO2) to fraction of inspired oxygen (FiO2) less than 300 mm Hg (PaO2 : FiO2), (3) respiratory rate greater than 30 breaths per minute, (4) lung infiltrates greater than 50% within 24 to 48 hours of hospital admission based on chest imaging, (5) respiratory failure, (6) septic shock, or (7) multiorgan dysfunction. Patients were excluded from the study if they had clinical concern for COVID-19 but negative SARS-CoV-2 by RT-PCR testing, did not receive convalescent plasma, or were less than 18 years of age.

### 2.4. Procedures

Eligible patients were allocated with the use of a web-based system (REDCap) with concealment of antibody concentration levels at baseline [16]. Information was recorded through chart review of the EMR on demographic data, level of respiratory support at the time of plasma infusion, length of hospital stay, subsequent receipt of mechanical ventilation in previously nonventilated patients, number of ventilator days, vital status, days from symptom onset to plasma infusion, and concomitant treatment with remdesivir, glucocorticoids, or both. The level of respiratory support was divided into those not requiring any oxygen, those receiving oxygen via nasal cannula, those receiving noninvasive ventilation or use of high-flow oxygen devices, and those receiving invasive mechanical ventilation or extracorporeal membrane oxygenation (ECMO).

The median concentrations of the quantitative measures for IgG and IgM antibodies in the convalescent plasma for the receptor-binding domain of the spike protein, the S1 subunit of the spike protein, and the nucleocapsid protein were determined and used as thresholds to designate high and low antibody concentration groups. Thus, eligible patients were assigned in a 1 : 1 ratio to receiving either high concentration convalescent plasma or low concentration convalescent plasma in addition to standard treatment. It should be noted that high and low concentration assignments were not consistent across all three domains. For example, plasma from a donor may have contained a high antibody concentration for the nucleocapsid protein, but the same plasma may have contained a low antibody concentration for the S1 subunit of the spike protein. Thus, the three antibody domains were analyzed separately with regard to outcomes. Patients were eligible if they received remdesivir, glucocorticoids, or both according to the standard of care at each institution. At the early phase at which this study was undertaken since the COVID pandemic began, the literature had been inconsistent about the efficacy of convalescent plasma infusion; therefore, finding an expected effect size in efficacy between low and high antibody concentration groups was not feasible. Thus, it was not possible to calculate a sample size to validate the expected percent improvement in outcomes. This study enrolled a convenient sample of 96 patients.

### 2.5. Outcome Measures

The primary outcome was all-cause mortality within 30 days following intervention. Secondary outcomes were length of hospital stay, number of ventilator days, and subsequent receipt of invasive mechanical ventilation (including extracorporeal membrane oxygenation) in patients who were previously not receiving mechanical ventilation. In addition, overall 30-day mortality based on the time from symptom onset to convalescent plasma infusion was measured. The number of ventilator days was recorded from EMR data during hospitalization. Information regarding the primary and secondary outcomes is complete for all study participants.

### 2.6. Statistical Analysis

Univariate analyses were used to (a) test for normality of continuous distributions prior to subjecting them to parametric tests and (b) describe the patient population in terms of demographics and quantitative measures of the transfused IgG and IgM antibody concentrations. Given the preponderance of nondetectable IgM concentrations, a missing value analysis was performed on the IgM antibody concentrations to look for covariates that may explain this lack of antibody detection. Frequency distributions of the antibody concentrations were also used to determine the medians of each IgG and IgM protein distributions above and below which became designates of high and low antibody concentrations for each of the antibody domains (RBD, nucleocapsid protein, and S1 subunit of the spike protein).

Bivariate *T*-test analyses were also used to test for statistical differences in the outcome measures between high vs. low concentrations of the Ab concentrations. For each antibody component, stratified Kaplan Meier analyses were performed to compare percent mortality in low vs. high antibody concentration groups using time since convalescent plasma infusion as the time-dependent variable. Significance of the % mortality over time between high vs. low antibody concentration groups was tested using the log-rank procedure. Statistically significant differences between low vs. high concentration groups were accepted at an alpha level of ≤0.05. Analyses were performed in IBM-SPSS ver 28.0, Chicago, IL.

## 3. Results

### 3.1. Patients

This retrospective cohort study consisted of 96 patients hospitalized at 3 hospitals in the Ascension Saint Thomas system, admitted between April 1, 2020, and July 31, 2020.  Table 1 shows key characteristics of the patients in the study. Overall, the mean (±SD) age of the patients in this study was 60.3 ± 15.4 years, 63.5% of the patients were men, 32.3% of the patients were black, and 28.1% of the patients were Hispanic or Latino. At the time of transfusion, 16.7% of patients were receiving mechanical ventilation or extracorporeal membrane oxygenation, 24% were receiving noninvasive ventilation or use of high-flow oxygen devices, 57.3% were requiring oxygen via nasal cannula only, and 2.1% were not receiving any oxygen. Of those who were previously nonventilated, 21.3% of patients subsequently received mechanical ventilation or extracorporeal membrane oxygenation. Overall, 56.3% of patients received treatment with IV steroids and 44.8% received treatment with remdesivir.

The median antibody concentrations to RBD, nucleocapsid protein, and S1 subunit domains were 8.25, 21.17, and 9.98 (the median antibody concentrations to RB*μ*g/mL), respectively. Across all three antibody domains, there were no significant differences in age, sex, race, body mass index, days from symptom onset, receipt of IV steroids, or receipt of remdesivir between low and high concentration groups. In addition, no significant differences were noted in the amount of respiratory support between low and high concentration groups across all three domains. None of the patients experienced a transfusion reaction.

### 3.2. Primary Objective

**Figure 1:**
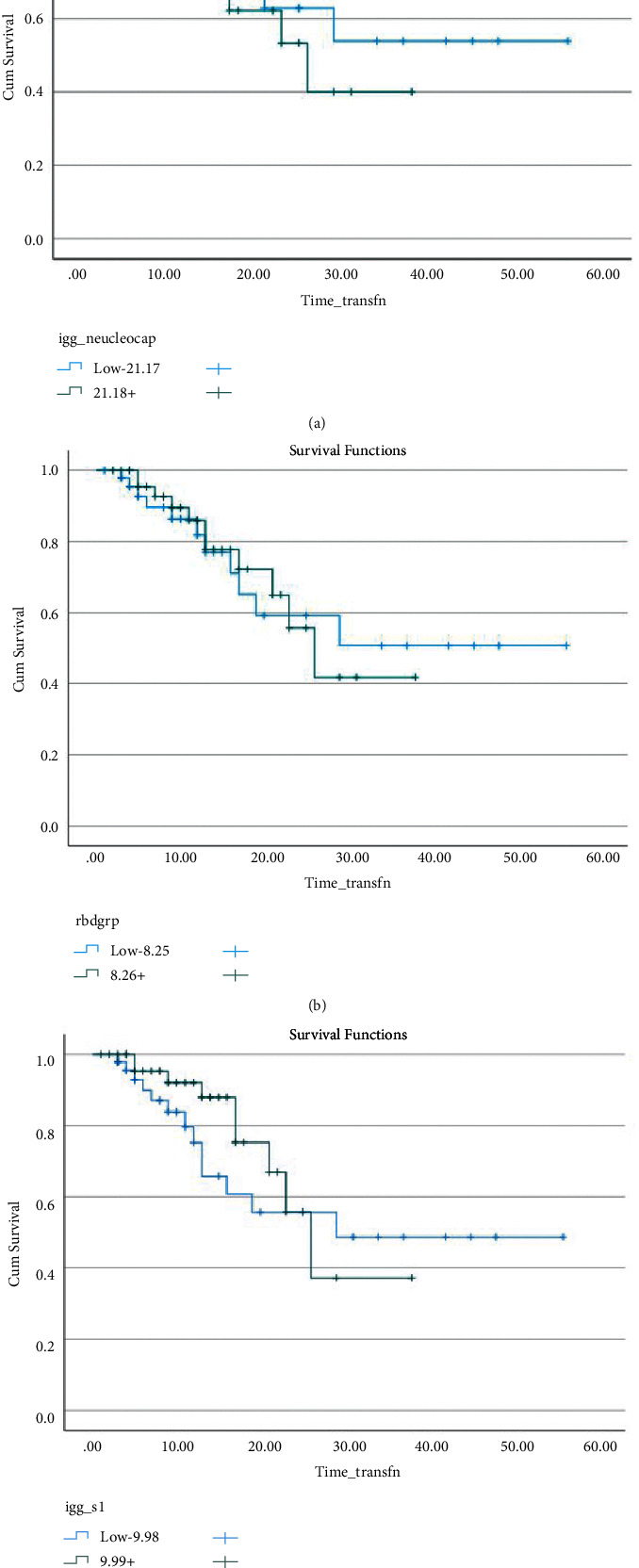
(a) Survival of patients since receiving COVID-19 convalescent plasma infusion: nucleocapsid protein domain. (b) Survival of patients since receiving COVID-19 convalescent plasma infusion: RBD domain. (c) Survival of patients since receiving COVID-19 convalescent plasma infusion: S1 subunit domain. (a) Kaplan–Meier estimates of the mean times to death following plasma transfusion within the nucleocapsid protein domain. Time since convalescent plasma transfusion is the time-dependent variable on the *x*-axis with cumulative survival plotted on the *y*-axis. Low antibody concentration data is represented in blue and high antibody concentration is represented in green. (b) Kaplan–Meier estimates of the mean times to death following plasma transfusion within the receptor-binding domain (RBD). Time since convalescent plasma transfusion is the time-dependent variable on the *x*-axis with cumulative survival plotted on the *y*-axis. Low antibody concentration data is represented in blue and high antibody concentration is represented in green. (c) Kaplan–Meier estimates of the mean times to death following plasma transfusion within the S1 subunit domain. Time since convalescent plasma transfusion is the time-dependent variable on the *x*-axis with cumulative survival plotted on the *y*-axis. Low antibody concentration data is represented in blue and high antibody concentration is represented in green.

At 30 days, death after plasma transfusion occurred in 22.9% of all of the patients (22 of 96 patients). Within the nucleocapsid antibody domain, the death event occurred in 10 patients in the low antibody concentration group versus 12 patients in the high antibody concentration group (22.2% versus 23.5%, *p*=0.88) (Table 2 and Figure 1(a)). Within the RBD antibody domain, death occurred in 11 patients in the low concentration group versus 11 patients in the high concentration group (22.9% versus 22.9%, *p*=1.0) (Table 2 and Figure 1(b)). The largest 30-day mortality benefit was seen within the S1 antibody domain, in which death occurred in 13 patients in the low concentration group versus 9 patients in the high concentration group (27.1% versus 18.8%, *p*=0.33) (Table 2 and Figure 1(c)). Thus, no significant differences were noted in 30-day mortality between high and low antibody concentration groups across all three antibody domains.

Although the Kaplan-Meier estimates of the mean times to death from plasma transfusion appear to be different between low vs. high antibody concentration groups for nucleocapsid (37.7 days vs. 25.3 days), RBD (35.9 days vs. 26.2 days), and S1 (34.2 days vs. 26.5 days), respectively, these comparisons were not found to be statistically significant as noted by the rank sum tests of >0.05 (Figures 1(a)–1(c) and Tables 3, 4, and 5).

### 3.3. Secondary Objectives

Patients in the high concentration groups across the three domains had shorter durations of hospital stays compared to the low concentration groups on average (Table 2), though there was considerable variability in the standard deviations in the low concentration groups and none of these comparisons reached statistical significance. The greatest difference in length of stay was seen with the nucleocapsid IgG high vs. low concentration (14.2 days vs. 18.3 days, resp., *p*=0.07). Of the 80 patients who were not receiving invasive mechanical ventilation at the time of convalescent plasma administration, 17 of these patients (21.3%) were subsequently mechanically ventilated. Among those 17 patients, the risk of progression to invasive mechanical ventilation was no different between low and high concentration groups across the three domains (*p* values of 0.61, 0.88, and 0.94 in the nucleocapsid, RBD, and S1 domains, resp.). Of the 33 patients who required invasive mechanical ventilation at any point during the study period, the number of days on the ventilator was noted to be shorter in the high concentration groups across all three domains. The absolute mean differences between high and low concentration groups in this outcome were 7.5, 6.1, and 5.5 days in the nucleocapsid, RBD, and S1 domains, respectively. None of the three domains reached statistical significance in this outcome. Lastly, 67 patients received plasma infusion less than 10 days from symptom onset compared to 29 patients who received infusion at or beyond 10 days. Between these two groups, 30-day mortality was lower in those patients who received plasma less than 10 days from symptom onset (20.9% versus, 27.6%, *p*=0.474). This did not reach statistical significance.

## 4. Discussion

In this retrospective cohort study of patients admitted to the hospital with SARS-CoV-2, we looked to evaluate the efficacy of convalescent plasma based on antibody concentration to three of the key antigens of the virus: the receptor-binding domain (RBD) of the spike protein, the S1 subunit of the spike protein, and the nucleocapsid (N) protein. Our results showed no statistically significant difference in 30-day mortality between high and low antibody concentration groups across all three domains after matching for demographic characteristics and respiratory requirements at the time of plasma transfusion. Among the three domains, the greatest mortality benefit was seen in the S1 subunit high antibody concentration group, with an observed 8.3% difference in mortality (18.8% vs. 27.1% in the high vs. low antibody concentration groups, resp., *p*=0.33). However, this study was not adequately powered to detect this difference.

In addition, no statistically significant difference was seen between high and low antibody concentration groups among the three domains in length of hospital stay. It should be noted that there was a trend for hospital length of stay being shorter in those who received high antibody concentration plasma, with mean absolute differences of 1.8, 2.5, and 4.1 days in the RBD, S1 subunit, and nucleocapsid domains, respectively. This trend in better outcomes for those in the high antibody concentration groups was also true for the number of ventilator days, with mean absolute differences of 6.1, 5.5, and 7.5 days in the RBD, S1 subunit, and nucleocapsid domains, respectively. There was no difference in the risk of progression to invasive mechanical ventilation between high and low concentration groups.

We evaluated the overall mortality benefit among all patients receiving convalescent plasma based on time from symptom onset to plasma transfusion, regardless of antibody level. Those who received convalescent plasma less than 10 days from symptom onset had a lower risk of death at 30 days compared to those who received plasma at or after 10 days from symptom onset (20.9% vs. 27.6% in the high vs. low antibody concentration groups, resp., *p*=0.474). This benefit likely represents the importance of early transfusion when the host antibody response is not as robust. Despite this mortality benefit, the difference was again not statistically significant due to our small sample size.

Our study was unique in that it aimed to evaluate differences in outcomes between patients receiving convalescent plasma based on antibody concentration. Our results are in contrast to a retrospective study of 3082 patients conducted by Joyner et al. in which 30-day mortality was lower in patients receiving high-titer convalescent plasma compared to those receiving low-titer plasma who had not received mechanical ventilation before transfusion (relative risk, 0.66; 95% confidence interval [CI], 0.48 to 0.91) [9]. This was particularly seen in patients who received plasma within 3 days after receiving a diagnosis of COVID-19 compared to those who received transfusions later in the disease course, again signifying the importance of early administration. This study ultimately led the FDA to authorize the use of high-titer convalescent plasma for the treatment of patients hospitalized with COVID-19. Comparatively, our study was not as adequately powered, which could account for the lack of statistical significance in our outcomes. In addition, our study utilized a quantitative method for measuring anti-SARS-CoV-2 IgG and IgM to three separate antigens of the virus, compared to a qualitative IgG chemiluminescent immunoassay based on the detection of IgG antibodies against a recombinant form of the SARS-CoV-2 spike subunit 1 protein.

Many other studies to date have looked to evaluate the efficacy of convalescent plasma versus placebo. The Randomised Evaluation of COVID-19 Therapy (RECOVERY) trial compared high-titer convalescent plasma to usual care alone and found no significant difference in 28-day mortality, no difference in the proportion of patients discharged within 28 days, and no difference between the percentage of patients who progressed to invasive mechanical ventilation in previously nonventilated patients [11]. The patient population in this study was different from ours, however, as the majority of patients were white (78%) compared to our cohort (41.7%) and fewer patients were receiving mechanical ventilation at the time of transfusion (5%) compared to our study (16.7%). The Convalescent plasma for COVID (ConCOVID) study also found no difference in mortality, hospital stay, or day 15 disease severity between plasma-treated patients and patients receiving standard of care [15]. Interestingly, this trial was halted early by the investigators when the baseline SARS-CoV-2 neutralizing antibody titers of participant plasma and convalescent plasma were found to be comparable, questioning the potential benefit of convalescent plasma for the study population. It should be noted, however, that the median time from symptom onset to plasma transfusion was 10 days (IQR 6–15). Our results add to the conclusions of these and other trials which suggest limited clinical benefit of convalescent plasma in severe COVID-19 [11–15].

One possible explanation for the observed limited clinical benefit of convalescent plasma in patients with severe COVID-19 is that the host inflammatory response is already robust in these individuals, thus limiting the neutralizing effects of convalescent plasma. Current literature evaluating the efficacy of high-titer convalescent plasma on disease progression in patients presenting in the outpatient setting with less severe COVID-19 infection have shown mixed results. Korley et al. conducted a randomized, single-blind trial of 511 patients aged 50 years or older being treated in an emergency department for COVID-19 symptoms to receive either high-titer convalescent plasma or placebo if they presented within 7 days of symptom onset [17]. The administration of convalescent plasma did not prevent disease progression, and five patients in the convalescent plasma arm died compared to one patient in the placebo group.

In contrast, Libster et al. conducted a randomized, double-blind, placebo-controlled trial of 160 patients comparing the use of high-titer convalescent plasma to placebo in older adult patients who presented within 72 hours after the onset of mild COVID-19 symptoms [18]. They found that early administration of high-titer convalescent plasma reduced the progression of COVID-19, though there was no mortality difference noted. In a more recent multicenter, double-blind, randomized, controlled trial, Sullivan et al. aimed to demonstrate the benefit of high-titer convalescent plasma administration in patients with SARS-CoV-2 infection compared with control plasma within 9 days after symptom onset in the outpatient setting [19]. The primary outcome of the study was COVID-19-related hospitalization within 28 days after transfusion, regardless of vaccination status or risk factors for disease progression. The primary outcome event was observed in 17 of 592 (2.9%) participants who received convalescent plasma compared to 37 of 589 (6.3%) participants who received control plasma. Of the 54 patients who were hospitalized, 53 of them were unvaccinated. As demonstrated in other trials, early transfusion (defined as ≤5 days after symptom onset in their subgroup analysis) appeared to show more favorable outcomes with a lower risk of hospitalization. Of note, the trial was conducted in a relatively young population (median age of 43 years) in which recovery rate is high and only 6.8% of the participants in this trial were 65 years of age or older.

Our study did have several limitations. First, our sample size was quite small at 96 patients, though this sample size was similar to other trials in the literature [14, 15]. This significantly limited our ability to detect any between-group differences despite a trend towards better outcomes in the high antibody concentrations groups, specifically in hospital length of stay and number of ventilator days. This small sample size also hindered our ability to perform subgroup analyses. Another limitation is that our study only evaluated patients with severe COVID-19, as defined in the Methods section. Thus, the conclusions of this study cannot be extrapolated to those with milder cases of COVID-19. Our study also did not account for patient-specific comorbidities that may have placed certain patients at higher risk for death. Another fact to be considered is some of these study patients were enrolled relatively early during the pandemic when less was known about the management of COVID-19, and it is unclear if this caused any interference with the results.

It should also be noted that the mean number of days from symptom onset to plasma transfusion was 8 days among all patients in this study, which is around the time that many patients with severe COVID-19 begin to clinically deteriorate. Thus, no conclusions can be made about the efficacy of high concentration plasma in patients receiving this treatment earlier in the disease course. As mentioned previously, the greatest survival benefit with convalescent plasma has been demonstrated when administered early in the disease course [9]. The strengths of our study were that patient characteristics, including level of respiratory support at the time of plasma transfusion, were similar between the two groups and represented all ethnicities. Time from symptom onset to plasma transfusion was also similar between high and low concentration groups, which does appear to be an important factor in outcomes. We were also able to use a new method of antibody quantitation which offers better precision compared to titering.

The most current World Health Organization (WHO) guidance published in December 2021 recommends against the use of convalescent plasma for patients with nonsevere COVID-19 [20]. For patients with severe or critical COVID-19, the WHO recommends not to use convalescent plasma, except in the context of a clinical trial. Though we do recognize that the findings in the literature to date have been inconsistent regarding the efficacy of high-titer convalescent plasma, we believe that further studies are necessary to firmly establish recommendations for future use. More focused studies evaluating early use in the outpatient setting in high-risk patients would be of great benefit, especially in the immunocompromised population. In addition, with the evolution of SARS-CoV-2 variants in communities, locally sourced convalescent plasma should theoretically contain antibodies with high neutralization capabilities to the most prevalent local viral strains which could prove to be more protective than current monoclonal antibody therapies to which the virus can become resistant [21]. Further research would be of great value for not only the current COVID-19 pandemic, but the future pandemics as well.

## 5. Conclusions

In conclusion, our analyses showed that, among patients who were hospitalized with severe COVID-19, convalescent plasma with high antibody concentration showed no significant difference in 30-day mortality, length of hospital stay, subsequent receipt of invasive mechanical ventilation, and number of ventilator days when compared to convalescent plasma with low antibody concentration. Further adequately powered research is necessary to evaluate the benefit of high antibody concentration convalescent plasma and which groups benefit the most. Future research evaluating early timing of plasma transfusion in high-risk patients would also be helpful, as there is evidence for better outcomes with early transfusion.

## Figures and Tables

**Table 1:** Characteristics of participants at baseline for each antibody domain based on antibody concentration.

Characteristic	All patients	Nucleocapsid protein			RBD			S1 subunit		
		High ab conc. (*N* = 51)	Low ab conc. (*N* = 45)	*p* values§	High ab conc. (*N* = 48)	Low ab conc. (*N* = 48)	*p* values§	High ab conc. (*N* = 48)	Low ab conc. (*N* = 48)	*p* values§
Age	60.3 ± 15.4	60.7 ± 14.1	59.9 ± 16.9	0.79	59.5 ± 15.4	61.2 ± 15.5	0.60	58.8 ± 14.7	61.9 ± 16.0	0.33
Male *N* (%)	61 (63.5)	36 (70.6)	25 (55.6)	0.13	32 (66.7)	29 (60.4)	0.53	33 (68.8)	28 (58.3)	0.29
Female *N* (%)	35 (36.5)	15 (29.4)	20 (44.4)		16 (33.3)	19 (39.6)		15 (31.3)	20 (41.7)	

Race—no. (%)^†^										
White	40 (41.7)	22 (43.1)	18 (40)	0.72	19 (39.6)	21 (43.8)	0.70	19 (39.6)	21 (43.8)	0.70
Black or African American	31 (32.3)	16 (31.4)	15 (33.3)		15 (31.3)	16 (33.3)		15 (31.3)	16 (33.3)	
American Indian/Alaskan native	1 (1)	1 (2)	0 (0)		1 (2.1)	0 (0)		1 (2.1)	0 (0)	
Asian	1 (1)	0 (0)	1 (2.2)		1 (2.1)	0 (0)		1 (2.1)	0 (0)	
Other^‡^	23 (24)	12 (23.5)	11 (24.4)		12 (25.0)	11 (22.9)		12 (25.0)	11 (22.9)	
BMI^††^	34.7 ± 10.7	33.8 ± 8.3	35.6 ± 12.8	0.43	33.6 ± 8.7	35.7 ± 12.4	0.35	34.0 ± 8.6	35.3 ± 12.5	0.58

Respiratory support at randomization, no. (%)				0.55			0.78			0.43
Not requiring oxygen	2 (2.1)	2 (3.9)	0 (0)		1 (2.1)	1 (2.1)		1 (2.1)	1 (2.1)	
Oxygen via nasal cannula only	55 (57.3)	29 (56.9)	26 (57.8)		30 (62.5)	25 (52.1)		30 (62.5)	25 (52.1)	
Noninvasive ventilation or use of high-flow oxygen devices	23 (24)	11 (21.6)	12 (26.7)		10 (20.8)	13 (27.1)		12 (25.0)	11 (22.9)	
Invasive mechanical ventilation or ECMO	16 (16.7)	9 (17.6)	7 (15.6)		7 (14.6)	9 (18.8)		5 (10.4)	11 (22.9)	

Days since symptom onset	8 ± 4	7.7 ± 3.8	8.7 ± 4.9	0.25	7.5 ± 3.7	8.9 ± 4.9	0.12	7.6 ± 3.6	8.8 ± 5.0	0.19
Coadministration of IV steroids during hospitalization, no. (%)	54 (56.3)	28 (54.9)	26 (57.8)	0.78	24 (50)	30 (62.5)	0.22	25 (52.1)	29 (60.4)	0.07
Coadministration of remdesivir during hospitalization, no. (%)	43 (44.8)	27 (52.9)	16 (35.6)	0.09	23 (47.9)	20 (41.7)	0.54	26 (54.2)	17 (35.4)	0.10

IgG concentration data, median (mean ± SD)¶										
RBD	8.25 (16.62 ± 19.68)									
Nucleocapsid protein	21.17 (24.58 ± 21.05)									
S1 subunit	9.98 (19.14 ± 23.13)									

^*∗*^Plus–minus values are means ± SD. §*p* values should be interpreted as summary statistics that quantify empirical variation of multilevel variables across multiple groups. They should not be interpreted as results of hypothesis tests. †Race was recorded in the patient's electronic health record. ‡The vast majority of these patients identified as Hispanic or Latino as their ethnicity. ††BMI was measured using kg/m^2^. ¶Antibody concentration was measured in *μ*g/mL.

**Table 2:** Primary and secondary outcomes.

Outcome	Nucleocapsid protein			RBD			S1 subunit		
	High ab conc. (*N* = 51)	Low ab conc. (*N* = 45)	*p* value	High ab conc. (*N* = 48)	Low ab conc. (*N* = 48)	*p* value	High ab conc. (*N* = 48)	Low ab conc. (*N* = 48)	*p* value
Primary outcome									
30-day mortality—no. (%)	12 (23.5)	10 (22.2)	0.88	11(22.9)	11(22.9)	1.00	9(18.8)	13(27.1)	0.33
Secondary outcomes									
Subsequent receipt of invasive ventilation in previously nonventilated—no. (%)	8/42 (19.0)	9/38 (23.7)	0.61	9/41 (22.0)	8/39 (20.5)	0.88	9/43 (20.9)	8/37 (21.6)	0.94
Length of hospital stay	14.2 ± 8.2	18.3 ± 13.3	0.07	15.2 ± 8.2	17.0 ± 13.3	0.42	14.9 ± 8.0	17.4 ± 13.4	0.26
Number of ventilator days (including those who subsequently received mechanical ventilation)	12.3 ± 8.11	19.8 ± 14.98	0.08	12.8 ± 8.3	18.9 ± 14.9	0.16	12.8 ± 8.3	18.3 ± 14.4	0.21

Overall mortality based on time to transfusion to death/end of cohort observation period									
30-day mortality based on time from symptom onset to plasma transfusion—no. (%)									
Infusion <10 days	14/67 (20.9)	*p*=0.474							
Infusion >/ = 10 days	8/29 (27.6)								

^*∗*^Plus–minus values are means ± SD.

**Table 3:** Means and medians for survival time for the nucleocapsid protein domain.

	Mean estimate	Std. error	95% confidence interval		Median estimate	Std. error	95% confidence interval	
			Lower bound	Upper bound			Lower bound	Upper bound
Nucleocapsid IgG low—21.17 *μ*g/ml	37.709	4.617	28.660	46.758	.	.	.	.
Nucleocapsid IgG 21.18 *μ*g/ml- high	25.285	2.707	19.979	30.591	26.000	5.961	14.317	37.683
Overall	35.328	3.445	28.576	42.081	29.000	.	.	.
			Chi-square		Df		Sig.	
Log rank (Mantel-Cox)			0.258		1		0.612	

Estimation is limited to the largest survival time if it is censored. Test of equality of survival distributions for the different levels of nucleocapsid IgG.

**Table 4:** Means and medians for survival time for the RBD domain

	Mean estimate % survival	Std. error	95% confidence interval		Median estimate	Std. error	95% confidence interval	
			Lower bound	Upper bound			Lower bound	Upper bound
RBD IgG low—8.25 *μ*g/ml	35.908	4.714	26.669	45.147	.	.	.	.
RBD IgG 8.26 *μ*g/ml—high	26.254	2.662	21.036	31.471	26.000	3.289	19.554	32.446
Overall	35.328	3.445	28.576	42.081	29.000	.	.	.
			Chi-square		Df		Sig.	
Log rank (Mantel-Cox)			0.018		1		0.892	

Estimation is limited to the largest survival time if it is censored. Test of equality of survival distributions for the different levels of RBD IgG.

**Table 5:** Means and medians for survival time for the S1 subunit domain.

	Mean estimate	Std. error	95% confidence interval		Median estimate	Std. error	95% confidence interval	
			Lower bound	Upper bound			Lower bound	Upper bound
S1 subunit IgG low—9.98 *μ*g/ml	34.242	4.531	25.362	43.122	29.000	.	.	.
S1 subunit IgG 9.99 *μ*g/ml—high	26.497	2.828	20.954	32.040	26.000	2.879	20.356	31.644
Overall	35.328	3.445	28.576	42.081	29.000	.	.	.
			Chi-square		Df		Sig.	
Log rank (Mantel-Cox)			0.746		1		0.388	

Estimation is limited to the largest survival time if it is censored. Test of equality of survival distributions for the different levels of S1 subunit IgG.

## Data Availability

Study data were collected and managed using REDCap electronic data capture tools hosted at Ascension. The individual level data collected/or analyzed during the study are not publicly available due to protection of participants privacy and confidentiality but are available from the corresponding author upon reasonable request.
